# *Salmonella* Serotyping Using Whole Genome Sequencing

**DOI:** 10.3389/fmicb.2018.02993

**Published:** 2018-12-13

**Authors:** George M. Ibrahim, Paul M. Morin

**Affiliations:** Microbiological Sciences Branch, Northeast Food and Feed Laboratory, United States Food & Drug Administration, Jamaica, NY, United States

**Keywords:** *Salmonella*, traditional serology, Kauffmann White scheme, whole genome sequencing, SeqSero

## Abstract

Until recently, traditional serology and the Kauffmann White Scheme (KWS) have been the gold standard for *Salmonella* serotyping. Whole Genome Sequencing (WGS) has now emerged as an alternative in this field. Serotype information remains a cornerstone in food safety and public health activities to reduce the burden of salmonellosis. At the same time, recent advances in WGS have improved the ability to perform advanced pathogen characterization while improving trace back investigations to determine the source of foodborne illness during outbreaks. Serovar prediction based on WGS can be performed using *in silico* data analysis tools. Three such tools have been developed: (a). *Salmonella in silico* Typing Resource (SISTR), (b). SeqSero, and (c). *in silico* 7-gene MLST ST (Multilocus Sequence Typing Sub-Typing) which was generated using the SISTR platform. Public health officials around the world are diligently working to validate these tools for replacing traditional surveillance methods to provide a more powerful approach for molecular epidemiology in support of public health investigations. In this study, we report a retrospective analysis of our laboratory inventory of 1,041 *Salmonella* isolates collected between 1999 and 2017. These isolates are of public health significance since they all came from either food, feed or environmental swabs. They were all serotyped by both traditional serology and WGS using an *in silico* SeqSero tool for serovar prediction. Both predicted identical *Salmonella* serotypes in 899 isolates (86.4% of the 1,041 *Salmonella* isolates). SeqSero assignments differed from traditional serological testing in 80 isolates (7.7%) and no serotype prediction was ascertained from 62 isolates (5.9%). This retrospective study is an excellent example of using WGS and SeqSero as a data analysis tool to predict *Salmonella* serotypes that can provide numerous advantages including molecular and genetic details regarding the characteristics of the *Salmonella* isolates compared to traditional KWS serotyping. In conclusion, it is evident that using WGS and *in silico* tools for *Salmonella* serotyping might someday replace traditional serotyping.

## Introduction

*Salmonella* was first visualized in 1880 by Karl Eberth. In 1884 George Theodor Gaffky successfully grew the pathogen in pure culture and in 1900 Joseph Leon Lignières proposed that the pathogen discovered by Daniel Salmon's group in 1885 be called *Salmonella* in his honor (Heymann et al., 2006). *Salmonella* has been known to cause 1.2 million foodborne illnesses annually in the United States with 23,000 hospitalizations and 450 deaths including many multistate outbreaks (CDC, 2018).

Although national surveillance data for *Salmonella* has been collected through laboratory-based surveillance systems based on serotype designation for over 50 years (Grimont and Weill, 2007), improvements in this surveillance system is desperately needed. It is crucial for public health and regulatory agencies to have more rapid, highly accurate, and discriminatory genomic and metagenomic serotyping methods to detect outbreaks and to link cases of illness to the originating source (Forbes et al., 2017).

Traditional *Salmonella* serotyping, according to the Kauffmann White Scheme (KWS) was developed in 1926 and has been used ever since as a proxy for genetics. It is maintained by the World Health Organization (WHO) Collaborating Centre for Reference and Research on *Salmonella*, located at the Pasteur Institute in Paris, France. The current (9th) edition issued in 2007 comprises antigenic variants that had been validated as of January 1, 2007. Currently there are 2,610 recognized *Salmonella* serovars (Dieckmann and Malorny, 2011).

The U.S. National *Salmonella* Surveillance System has been built upon serotyping in public health laboratories, a subtyping method performed through the agglutination of *Salmonella* cells with specific antisera that detect lipopolysaccharide O antigen and flagellar H antigens. Specific combinations of O and H antigenic types represent distinct serotypes or serovars (Samuel and Reeves, 2003).

Serovars are distinctive groups within a single species of microorganisms as bacteria and viruses which share common distinctive surface structures. In *Salmonella*, these surface structures are the O and H antigens. The O antigens are made of lipopolysaccharides which differ in their chemical constitution. The H antigens are slender threadlike structures which are parts of the flagella and are different in their protein content. Each O and H antigen has a unique code number and the scientists use their combinations to classify *Salmonella* bacteria that look similar under the microscope into many serotypes. Some serotypes are only found in one kind of animal or in a single place. Others can be found in many different animals and all over the world. Some can cause severe illness while others may only cause mild illness when they infect people. Public health scientists use serotyping to detect *Salmonella* outbreaks and to track them to their sources. All clinical strains of *Salmonella* are subtyped using molecular tools as well as being serotyped and when one molecular subtype increases above normal occurrence, an outbreak is suspected (CDC, 2015).

Molecular assays for serotyping of bacterial isolates are often developed on the Luminex platform to determine serotypes based on DNA markers within genes responsible for O and H antigen expression (Vanegas and Joys, 1995; Fitzgerald et al., 2007). The Luminex xMAP technology allows detection of nucleic acids by combining PCR with a multiplexed bead-based detection system. The antigen that is encoded by the PCR fragment is identified by hybridizing with antigen specific-DNA probes. Although it is fairly rapid and not prohibitively expensive when one is looking at specific or common serotypes, the one current limiting factor of this technology is that the molecular serotyping does not detect all serotype antigens and focuses primarily on the most common serotypes reported for human clinical specimens (McQuiston et al., 2004, 2011).

Next generation sequencing technology provides lots of information about species, serovar, virulence, pathogenicity, antimicrobial resistance, and subtype of bacteria in just one sequencing test. WGS can generate high-quality sequence data in public health laboratories with better identification of clinical strains relating them to outbreak strains with better determination of virulence and antimicrobial-resistance genes. WGS has already dramatically changed the field of genomics and could enable researchers to study gene expression by sequencing RNA and examining host-pathogen interactions (Oakeson et al., 2017).

WGS can also be used to identify the path of disease transmission within a population and provide information on the probable source. It is essential for mutation detection and in understanding of genetics of *Salmonella* and other microorganisms. It can also evaluate the evolution of strains during an outbreak and detect contextual data on the genetic interrelatedness (Gilchrist et al., 2015). A major application for WGS is to identify outbreak clusters and efficiently infer phylogenies from the sequencing reads (Ahrenfeldt et al., 2017).

WGS is used now as an alternative technique for obtaining fast and reliable serotype information (Gymoese et al., 2017). In this study we evaluate the potential use of WGS to serve as the sole method for the routine serotyping of *Salmonella* isolates. This approach offers a rapid identification of *Salmonella* serotypes as well as, identifying an array of single nucleotide polymorphisms (SNP) within the genome. These SNPs can be used to investigate the epidemiology of an outbreak to link human cases of illness to the point source of contamination and differentiate between outbreak-related and unrelated sporadic clinical cases (Lienau et al., 2011). WGS also has the potential to provide researchers and clinicians with additional information regarding antibiotic resistance markers and virulence factors to better understand serotypes and to quickly identify, and investigate outbreaks while providing trace-back and trace-forward information (Inns et al., 2015; Taylor et al., 2015).

WGS is rapidly replacing current molecular subtyping methods for surveillance and for foodborne outbreak purposes (Bakker et al., 2011; Leekitcharoenphon et al., 2014). It enables high-resolution molecular subtyping and provides valuable additional data regarding further characterization of emerging clones based on genetic differences and evolutionary studies (Bale et al., 2016; Bekal et al., 2016). This information is critical during any outbreak response for gathering clonal information in outbreak investigations. Several studies have shown that WGS-based typing has an enhanced discriminatory power compared to current molecular typing methods used for *Salmonella* (Koser et al., 2012; Deng et al., 2015). Public health microbiology is currently being transformed by next generation sequencing, which opens the door to more rapid serotype determination using WGS data. SeqSero (www.denglab.info/SeqSero) is a novel web-based tool for determining *Salmonella* serotypes using whole genome sequencing data (Zhang et al., 2015).

## Materials and Methods

### Study Design

A total of 1,041 *Salmonella* isolates from foods, feed and environmental samples were derived from our laboratory inventory between 1999 and 2017. All isolates were previously serotyped using traditional serology as well as having their genome sequenced. We used the online tool, SeqSero to evaluate whether traditional serology could be replaced with WGS and such online tool.

Metadata was collected for each *Salmonella* isolate which included a WGS accession ID number, an FDA tracking number, a date that our laboratory recorded the result in our internal database, and the date when the traditional serology was uploaded within our internal database. The metadata also included the results summary, traditional serotyping results, the WGS run date, flow cell bar code number, base calls and the predicted *Salmonella* serotype for every isolate.

### Genome Sequencing

WGS analysis starts by extraction of genomic deoxyribonucleic acid (DNA) with a fully automated Qiagen QIAcube™ from 1 mL of bacterial culture grown overnight in Brain Heart Infusion (BHI) broth at 35°C as per the FDA GenomeTrakr protocol. DNA is quantitated, fragmented and tagged with adapter sequences added to the ends (Nextera XT Library Prep Kit, Illumina, Inc.). The library is amplified by PCR followed by cleanup and size selection using AMPure XP™ (Beckman Coulter, Inc.) beads to remove very short library fragments from the sample. Library normalization is performed using the Nextera kit to ensure equal library representation in the pooled sample. The pooled amplicon library (PAL) is performed by combining equal volumes of normalized library, quantitated, and diluted in hybridization buffer forming a diluted amplicon library (DAL). This DAL is heat denatured and loaded onto the MiSeq reagent cartridge for sequencing^1^.

### Serovar Prediction From WGS Using *in silico* SeqSero Tool

SeqSero, was used to determine the serotype of the 1,041 *Salmonella* isolates. Paired reads of WGS raw data (fastq files) were uploaded to the online SeqSero tool version 1.0. The data input and output to and from SeqSero database predicted the *Salmonella* serotype of the requested isolate within a few minutes.

### Interpretation of Results

In order to calculate the number of days it took to complete the traditional serotyping, we subtracted the date on which our laboratory recorded the result in the FDA tracking database from the date of traditional serology results uploaded in the FDA tracking database for each isolate. We then reported the mean number of days it took to achieve traditional serology.

The results from the traditional *Salmonella* serotyping and from the WGS serotyping using SeqSero tool were analyzed for their differences and similarities, as well as their antigenic nomenclature.

## Results

### Description of Entire Cohort

We performed whole genome sequencing on 1,041 *Salmonella* isolates from our laboratory stock inventory and used the SeqSero database for predicting the serotype. SeqSero could not assign 62 isolates and the other 979 isolates contained 160 different *Salmonella* serovars, of which, *S*. Weltevreden was the most predicted serotype being assigned to 113 isolates of the 979 isolates (11.54%). Second in frequency was *S*. Typhimurium in 62 isolates (6.33%). *S*. Virchow came next being predicted in 50 isolates (5.11%). *S*. Senftenberg or *S*. Dessau which share the same general formula: 1,3,19:g,s,t:- was predicted in 45 isolates (4.6%). *S*. Tananarive and *S*. Brunei which share the same general formula: 8:y:1,5 were predicted in 32 isolates (3.27%). *S*. Infantis was found in 31 isolates (3.17%). *S*. Kentucky and *S*. Newport came next in frequency, each being predicted in 30 isolates (3.06%). *S*. Bardo was reported by SeqSero to share the same general formula as *S*. Newport, however, Bardo is very rare. *S*. Mbandka came next in frequency and was predicted in 29 isolates (2.96%). *S*. Paratyphi B or *S*. potential monophasic variant of Paratyphi B was predicted in 27 isolates (2.76%). Supplementary Table 1 shows all 160 serovars and their abundance.

In comparing the predicted serotypes based on WGS and SeqSero tool against the traditional *Salmonella* serology (KWS), both SeqSero (WGS) and traditional serotyping predicted identical *Salmonella* serotypes in 899 isolates (86.4% of the 1,041 *Salmonella* isolates). No serotype prediction was ascertained from 62 isolates (5.9% of the 1,041 *Salmonella* isolates) where SeqSero's result was “N/A (The predicted antigenic profile does not exist in the White-Kauffmann-LE Minor Scheme)” as shown in Supplementary Table 2. They will decrease overtime as more whole genomes are added to the *Salmonella* national database (NCBI). SeqSero assignments differed from traditional serological testing in 80 isolates (7.7% of the 1,041 *Salmonella* isolates) as shown in Table 1.

**Table 1:** Examples of *Salmonella* strains with different outcomes between traditional serotyping and SeqSero prediction.

**Traditional predicted *Salmonella* serotype and its antigenic formula**	**SeqSero predicted serotype of same isolate and its antigenic formula**
*Salmonella* Poona Group G1 1 13,22z1,6	*Salmonella* Oranienburg 6,7,14 m,t [z57]
*Salmonella* Zigong Group I 16 1,w 1,5	*Salmonella* Mbandaka 6,7,14 z10 e,n,z [z37,[z45]
*Salmonella* Weltevreden E1 3,{10} {15} r z6	*Salmonella* Zigong 16 1,w 1,5
*Salmonella* Anatum Group E1 3,{10}{15}{15,34} e,h 1,6	*Salmonella* Richmond 6,7 y 1,2
*Salmonella* Anatum Group E1 3,{10}{15}{15,34} e,h 1,6	*Salmonella* Caracas [1],6,14,[25] g,m,s -
*Salmonella* Newport Group C2 6,8,20 e,h 1,2	*Salmonella* Abony 1,4,[5],12,27 b e,n,x
*Salmonella* Saintpaul Group B 1,4,[5],12 e,h 1,2	*Salmonella* Braenderup 6,7,14 e,h e,n,z15
*Salmonella* Weltevreden Group E1 3,{10}{15} r z6	*Salmonella* Kirkee 17 b 1,2
*Salmonella* Saintpaul Group B 1,4,[5],12 e,h 1,2	*Salmonella* Mgulani 38 i 1,2
*Salmonella* Adelaide Group O 35 f,g – Z27	*Salmonella* Zigong 16 1,w 1,5
*Salmonella* Schwarzengund Group B 1,4,12,27 d 1,7	*Salmonella* Stanley 1,4,[5],12,27 d 1,2
*Salmonella* Montevideo Group C1 6,7,14,[54] g,m,[p],s [1,2,7]	*Salmonella* Rissen 6,7,14 f,g -
*Salmonella* Houten Group U II 43:z4,z23:-	*Salmonella* Farmingdale 43 z4,z23 [1,2]
*Salmonella* Saintpaul Group B 1,4,[5],12 e,h 1,2	*Salmonella* Ealing 35 g,m,s -
*Salmonella* Tennessee Group C1 6,7,14 Z29 [1,2,7]	*Salmonella* Senftenberg or Dessau (Share the same antigenic profile) 1,3,15,19 g,s,t: -
*Salmonella* Sandiego Group B 1,4,[5],12 e,h e,n,z15	*Salmonella* Chester 1,4,[5],12 e,h e,n,x
*Salmonella* Paratyphi B Group B 1,4,[5],12 b 1,2 [z5],[z33]	*Salmonella* Senftenberg or Dessau (Share the same antigenic profile) 1,3,19:g,s,t:-
*Salmonella* Nashua Group M 28 1,v e,n,z15	*Salmonella* Vitkin 28 1,v e,n,x
*Salmonella* Omuna Group C1 6,7 z10 z35	*Salmonella* Tamilnadu 6,7 z41 z35
*Salmonella* Itami Group D1 9,12 1,z13 1,5	Salmonella Javiana or II 9,12:I,z28:1,5 (Share the same antigenic profile)

### SeqSero Could Predict 36 Isolates When Traditional Serotyping Could Not

Among the 899 isolates, 36 isolates were only predicted as monophasic, diphasic or non-motile by traditional serology without any further speciation, while SeqSero analysis provided the strain name of these same isolates. For example an isolate assigned as *Salmonell*a Saintpaul by SeqSero, was only serotyped as *Salmonella* Monophasic Group B by traditional *Salmonella* serology. Another example was an isolate serotyped as *Salmonella* Monophasic Group C1 by traditional serology but assigned as *Salmonella* Virchow by SeqSero, as shown in Table 2.

**Table 2:** Examples of isolates in which traditional serology predicted monophasic or non-motile *Salmonella* while WGS was able to predict *Salmonella* serotypes as nominated in KWS.

**Traditional predicted *Salmonella* serotype**	**SeqSero (WGS) predicted serotype of same isolates**
*Salmonella* Monophasic Group B	*Salmonella* Saintpaul 1,9,12 a 1,7
*Salmonella* Monophasic Group C1 (Antigenic formula 6,7,14:-:1,2)	*Salmonella* Virchow 6,7,14 r 1,2
Non-Motile *Salmonella* Group C1	*Salmonella* Mbandaka 6,7,14 z10 e,n,z15 [z37][z45]
*Salmonella* Monophasic Group D1	*Salmonella* Senftenberg or Dessau 1,3,19:g,s,t:-
Monophasic *Salmonella* Group I (Antigenic formula = 16: b:-)	*Salmonella* Weltevreden 3,{10}{15} r z6
Monophasic *Salmonella* Group B	*Salmonella* potential monophasic variant of Paratyphi B 1,4,[5],12 b 1,2 [z5],[z33]
*Salmonella* Group C2. No Flagellar Antigens.	*Salmonella* Tananarive 6,8 y 1,5 or Brunei 8,20 y 1,5
*Salmonella* Group C2 (Antigenic formula = ssp I 8:-: 1,5)	*Salmonella* Enteriditis 1,9,12 g,m -
Salmonella Monophasic Group C2	*Salmonella* Tananarive 6,8 y 1,5 or Brunei 8,20 y 1,5
*Salmonella* ssp. 16, 7:y:1,2,5 Group C1	*Salmonella* Bareilly 6,7,14 y 1,5
*Salmonella* Monophasic Group C1	*Salmonella* Thompson 6,7,14 k 1,5 [R1…]
*Salmonella* Monophasic Group B (Antigenic Formula = 4, 5:i-)	*Salmonella* Typhimurium(O5-) 1,4,[5],12 I 1,2
*Salmonella* Monophasic Group B (Antigenic Formula 4, 5: i:-)	*Salmonella* potential monophasic variant of Typhimurium 1,4,[5],12 I 1,2
*Salmonella* Monophasic Group G2 (Antigenic formula = 13,23:z:-)	*Salmonella* Farmsen 13,23 z 1,6 or Poona 1 13,22z1,6
*Salmonella* Group H	*Salmonella* Augustenborg 6,7.14 i 1,2
*Salmonella* Non-Motile Group B	*Salmonella* Tananarive 6,8 y 1,5 or Brunei 8,20 y 1,5
*Salmonella* Monophasic Group I	*Salmonella* Butantan 3,{10}{15}{15,34} b 1,5
*Salmonella* Monophasic Group C2	*Salmonella* Hindmarsh 8,20 r 1,5 or Bovismorbificans 6,8,20 r,[i] 1,5
*Salmonella* Group J. ssp I (17:I:-)	*Salmonella* Idikan 1,13,23 i 1,5
*Salmonella* ssp. 1 Group C1. Unable to further serotype	*Salmonella* Infantis 6,7,14 r 1,5 [R1…],[Z37],[Z45],[Z49]
*Salmonella* Monophasic Group C1	*Salmonella* Thompson 6,7,14 k 1,5 [R1…]
*Salmonella* Group B (Antigenic formula = 4,5,12:b:-)	*Salmonella* monophasic variant of Paratyphi B 1,4,[5],12 b 1,2 [z5],[z33]
Non-motile *Salmonella* Group C2	*Salmonella* Tananarive 6,8 y 1,5 or Brunei 8,20 y 1,5
*Salmonella* Monophasic Group G2	*Salmonella* Farmsen 13,23 z 1,6 or Poona 1 13,22z1,6
*Salmonella* 4, 12: b:- Group B	*Salmonella* potential monophasic variant of Paratyphi B 1,4,[5],12 b 1,2 [z5],[z33]
Monophasic *Salmonella* Group D1. (Antigenic formula = 1, 9, 12:-:1, 5)	*Salmonella* Javiana 1,9,12 1,z28 1,5 [R…]
*Salmonella* Monophasic gr B. (Antigenic formula = 1,4,5,12:-:1,2,7)	*Salmonella* Typhimurium 1,4,[5],12 I 1,2

### SeqSero Provided More Molecular Details Regarding *Salmonella* Typhimurium

Traditional serology predicted it as *Salmonella* Typhimurium in 62 isolates, while SeqSero was able to further detect the absence of the O5 epitope in 20 of these isolates.

### SeqSero Assigned Two Possible Serotypes Sharing the Same Antigenic Profile

SeqSero analysis of 105 isolates provided two possible serotypes sharing the same antigenic profile (or formula) but differed in minor O antigenic factors. Traditional serotyping predicted only one serotype from each of these isolates which was in agreement with one of the two SeqSero calls. For example, traditional serology predicted some isolates as *Salmonella* Brunei and these same isolates were assigned by SeqSero as *Salmonella* Tananarive or Brunei since both share the same general formula of “8:y:1,5.” Other isolates which were predicted as *Salmonella* Senftenberg by traditional serotyping, were assigned as *Salmonella* Senftenberg or Dessau by SeqSero because both share the same general formula “1,3,19:g,s,t:-” as shown in Table 3.

**Table 3:** Examples of *Salmonella* strains sharing the same general formula as assigned by SeqSero vs. traditional serology, which predicted only one serotype.

**Traditional predicted serotype**	**SeqSero (WGS) predicted serotypes**	**Shared general formula**
*Salmonella* Brunei	*Salmonella* Tananarive or Brunei	8:y:1,5
*Salmonella* Blockley	*Salmonella* Haardt or Blockley	8:k:1,5
*Salmonella* Senftenberg	*Salmonella* Senftenberg or Dessau	1,3,19:g,s,t:-
*Salmonella* Kottbus	*Salmonella* Kottbus or Ferruch	8:e,h:1,5
*Salmonella* Emek	*Salmonella* Chincol or Emek	8:g,m,s:-
*Salmonella* Albany	*Salmonella* Albany or Duesseldorf	8:z4,z24:-
*Salmonella* Corvallis	*Salmonella* Corvallis or Chailey	8:z4,z23:-
*Salmonella* Farmsen	*Salmonella* Farmsen or Poona	13:z:1,6
*Salmonella* Bovismorbificans	*Salmonella* Hindmarsh or Bovismorbificans	8:r:1,5
*Salmonella* Muenchen	*Salmonella* Virginia or Muenchen	8:d:1,2
*Salmonella* Lichfield	*Salmonella* Pakistan or Litchfield	8:l,v:1,2
*Salmonella* Sundsvall	*Salmonella* Soahanina or Sundsvall	6,14:z:e,n,x
*Salmonella* Telelkebir	*Salmonella* Diguel or Telelkebir	13:d:e,n,z15
*Salmonella* Madelia	*Salmonella* Carrau or Madelia	6,14:y:1,7
*Salmonella* Miami	*Salmonella* Miami or Sendai or II 9,12:a:1,5	9:a:1,5
*Salmonella* Glostrup	*Salmonella* Glostrup or Chomedey	8:z10:e,n,z15
*Salmonella* Cubana	*Salmonella* Agoueve or Cubana	13:z29:-
*Salmonella* Hadar	*Salmonella* Hadar or Istanbul	8:z10:e,n,x
*Salmonella* Kintambo	*Salmonella* Kintambo or Washington	13:m,t:-

Traditional serotyping determined some isolates to be *Salmonella* Newport and although SeqSero assigned them as the same designation, SeqSero also noted that *Salmonella* Bardo shares an identical antigenic profile however Bardo is an exceedingly rare serotype across the globe (Gupta et al., 2016).

## Discussion

### Limitations of Traditional Serotyping

The cost of performing traditional serotyping can be exorbitant. The price of 3 ml antisera vial varies from $87 to $267, depending on the type of poly group. For instance, in 2017 NFFL spent $4,000 to purchase poly A to poly G antisera for preliminary serotyping that is required for identification and confirmation of *Salmonella* isolates. Then, these isolates were shipped to a central laboratory where serovar (phenotype) identification was performed. The weekly shipping cost was $350, this rate remained the same regardless of the number of isolates in the shipping container. Overall, the budget required for traditional *Salmonella* serotyping including shipping was ~$20,000 with increasing costs each year.

The phenotypic determination of isolates in the central laboratory is labor-intensive and time-consuming with a turnaround time between 1 and 3 weeks depending on the isolate being processed. Some isolates require several passes through semi-solid media to enhance motility and flagellar antigenic expression (CDC, 2015) and some do not express serotype antigens due to a single nucleotide change in the genome (Li et al., 2017) thus, limiting the utility of traditional serotyping. Traditional serotyping requires a large number of tubes and slides to complete the whole process, which requires large laboratory space and may compromise quality control, as maintenance of all the necessary reagents can be difficult when managing a full array of antisera.

### Using SeqSero Tool to Predict *Salmonella* Serotypes

WGS has been recently used in the field of *Salmonella* subtyping and may ultimately prove to be more reliable and efficient. SeqSero is a web-based tool, which can predict most *Salmonella* serotypes using high-throughput genome sequencing data based on the databases of *Salmonella* serotype determinants. SeqSero extracts the relevant genomic regions, specifically the *rfb* gene cluster for somatic antigen determination and the *fliC* and *fljB* alleles for the H1 and H2 antigens, from the genome assemblies and aligns these regions to curated databases. The performance of SeqSero was evaluated by testing (i) raw reads from genomes of 308 *Salmonella* isolates of known serotype; (ii) raw reads from genomes of 3,306 *Salmonella* isolates sequenced and made publicly available by GenomeTrakr, a U.S. national monitoring network operated by the Food and Drug Administration; and (iii) 354 other publicly available draft or complete *Salmonella* genomes. *Salmonella* serotype determination from raw sequencing reads of fecal metagenomes from mice which were orally infected with this pathogen were also demonstrated (Zhang et al., 2015).

### Limitations of Whole Genome Sequencing

Further considerations must also be given to the costs associated with sequencing, the technical and informational capacities of national and partner laboratories, turnaround times associated with the batching of isolates, and data sharing models to improve the flow of information between various partners (Alkan et al., 2011).

SeqSero gave some incorrect results due to incorrect calling of various antigenic determinants, especially in regards to closely related serovars, such as those that differ on the basis of flagellar antigens of the g-complex. SeqSero analysis of some isolates provided two possible serotypes sharing the same antigenic profile (or formula) but differed on minor O antigenic factors. SeqSero could not predict some isolates. These unpredicted serovars are either rare *Salmonella* isolates or possibly there were some gaps within the SeqSero database.

### Advantages of Using SeqSero Tool For Serovar Prediction

Although *in silico* serotyping may have some limitations, our study shows that whole genome sequencing used with the SeqSero tool can have many advantages that far out way the limitations. It proved to be a very fast and efficient way to accurately predict *Salmonella* serotypes. By using the SeqSero database with the raw whole genome sequencing, we were able to accurately predict serotypes for *Salmonella* isolates with as much accuracy (or better) as that provided by the traditional KWS protocols. We found that successful results were obtained for 899 (86.4%) isolates having the same antigenic formula and serotype call. This percentage can only be expected to increase over time with the addition of new strains to the national database and improvements made to the SeqSero algorithm. This study shows the overall suitability of replacing traditional phenotypic methods with genomic serotyping using the SeqSero tool.

While both SISTR and MLST also provide us with increased phylogenetic classification which can be used to answer additional epidemiological questions; SeqSero provides the opportunity to analyze results directly from raw reads (Yachison et al., 2017).

In addition, SeqSero provided a wealth of genomic details that can be very important in determining *Salmonella* phylogenetic associations or understanding nuances of pathogenic differences. At the same time, future investigational studies on the isolates that did not yield matching results between SeqSero and traditional serotyping will be performed.

One example of SeqSero providing useful pathogenic information was discovered in 20 *Salmonella* Typhimurium isolates that were found to be absent of the O5 epitope. This epitope is absent due to a frameshift deletion detected in the *oafA* gene mutation which can have a significant role in complement-mediated killing of bacteria (Fox et al., 2005).

Routine and real-time implementation of WGS has the potential to transform public health microbiology (Joensen et al., 2014). Efforts have been made to enable a variety of pathogen subtyping and characterization analyses through the use of WGS data, such as multi-locus sequence typing (Inouye et al., 2012; Larsen et al., 2012), antimicrobial resistance identification, and virulence characterization (Zankari et al., 2012). Beyond WGS of pure cultures, recent application of metagenome sequencing in diagnosis and outbreak investigation of infectious diseases has demonstrated the potential for culture-independent detection of pathogens from complex clinical samples (Loman et al., 2013). For *Salmonella* serotyping, databases were built for O and H antigen determination (Jiang et al., 1991). The significant use of WGS technology has clearly been demonstrated in epidemiological studies and outbreak detection for *Salmonella* and other enteric pathogens revealing outbreak associations missed by standard Pulsed-Field Gel Electrophoresis (Den Bakker et al., 2014; Scaltriti et al., 2015).

Our laboratory routinely uses WGS for predicting *Salmonella* serotypes for every isolate from current investigations (food/feed/environmental) and from our archived isolates from previous years. Given our results, serotyping based on WGS prediction using SeqSero can be widely used as reliable method for *Salmonella* serotyping due to its numerous advantages of accurate and more granular details describing the molecular characteristics of *Salmonella* isolates. Furthermore, major budgetary savings as well as expeditious result times compared to the traditional serotyping using the KWS or molecular serotyping methods are other significant advantages for SeqSero serotyping.

## Conclusion

It is now routine for our laboratory to perform whole genome sequencing and traditional serotyping for every real time *Salmonella* isolate from animal feed, human foods, and environmental swabs. Using WGS and the *in silico* SeqSero tool as the sole method for *Salmonella* serotyping can have numerous advantages as it allows for very quick and accurate serotype predictions, detailed genetic information (antimicrobial resistance and virulence), significant budgetary savings, and reduced labor requirements. As more whole genomes are added daily to the NCBI *Salmonella* national database, the ability of SeqSero and online databases to make accurate assignments of *Salmonella* serotypes will continue to improve and expand.

## Author Contributions

GI designed study, collected data, performed statistical analysis, analyzed data, and main contributor to manuscript writing. PM assisted with study design and critically revised intellectual content of the manuscript.

### Conflict of interest statement

The authors declare that the research was conducted in the absence of any commercial or financial relationships that could be construed as a potential conflict of interest.

## Footnotes

^1^Methods/Pulsenet/CDC, W. G. S. W. P, and Available online at: www.cdc.gov/pulsenet/pathogens/wgs.html. February 11.
