# Supplementary material for: Salmonella Serotyping Using Whole Genome Sequencing
Source: Front Microbiol. 2018 Dec 13;9:2993. doi: 10.3389/fmicb.2018.02993 (PMC6300517; doi:10.3389/fmicb.2018.02993)
Supplement: Supplementary file 1 [file Table_1.PDF]

Supplementary Table 1

Serovars serotyped in 1041 isolates:

| | ***Salmonella* serovar and its antigenic formula** | **Number of isolates** |
|---|---|---|
| 1 | S. Weltevreden 3,{10}{15} r $z_{6}$ | 113 |
| 2 | S. Typhimurium 1,4,[5],12 i 1,2 and S. Typhimurium(O5-) | 62 |
| 3 | S. Virchow 6,7,14 r 1,2 | 50 |
| 4, 5 | S. Senftenberg or S. Dessau (Share the same general formula: 1,3,19:g,s,t:- ) | 45 |
| 6,7 | S. Tananarive or S. Brunei (Share the same general formula: 8:y:1,5) | 32 |
| 8 | S. Infantis 6,7,14 r 1,5 | 31 |
| 9,10 | S. Newport or S. Bardo (Share the same general formula but is very rare)<br>S. Newport 6,8,20 e,h 1,2 [$z_{67}$],[$z_{78}$] S. Bardo 8 e,h 1,2 | 30 |
| 11 | S. Kentucky 8,20 i $z_{6}$ | 30 |
| 12 | S. Mbandka 6,7,14 $z_{10}$ e,n,$z_{15}$ | 29 |
| 13 | S. Paratyphi B or S. potential monophasic variant of Paratyphi B 1,4,[5],12 b 1,2 | 27 |
| 14 | S. Gaminara 16 d 1,7 | 23 |
| 15 | S. Enteritidis 1,9,12 g,m - | 22 |
| 16 | S. Saintpaul 1,4,[5],12 e,h 1,2 | 17 |
| 17 | S. Bareilly 6,7,14 y 1,5 | 17 |
| 18 | S. Stanley 1,4,[5],12,27 d 1,2 | 17 |
| 19 | S. Agona 1,4,[5],12 f,g,s [1,2] [$z_{27}$],[$z_{45}$] | 16 |
| 20 | S. Javiana 1,9,12 1,z28 1,5 [R1…] | 16 |
| 21 | S. Anatum 3,{10}{15}{15,34} e,h 1,6 [$z_{64}$] | 14 |
| 22 | S. Lexington 3,{10}{15}{15,34} $z_{10}$ 1,5 [$z_{49}$] | 14 |
| 23 | S. Montevideo 6,7,14,[54] g,m,[p],s [1,2,7] | 14 |
| 24 | S. Thompson 6,7,14 k 1,5 [R1…] | 14 |
| 25 | S. Havana 1,13,23 f,g[s] – [z79] | 12 |
| 26 | S. Schwatzengrund 1,4,12,27 d 1,7 | 12 |
| 27 | S. Meleagridis 3,{10}{15}{15,34} e,h 1,w | 11 |
| 28 | S. Orion 3,{10}{15}{15,34} y 1,5 | 10 |
| 29 | S. Derby 1,4,[5],12 f,g [1,2] | 10 |
| 30 | S. Aberdeen 11 i 1,2 | 10 |
| 31 | S. Orion 3,{10}{15}{15,34} y 1,5 | 10 |
| 32 | S. Oranienburg 6,7,14 m,t [$z_{57}$] | 9 |
| 33,34 | S. Poona or S. Farmsen (share the same general formula: 13:z:1,6 ) | 9 |
| 35 | S. Bredeney 1,4,12,27 1,v 1,7 [$z_{40}$] | 8 |
| 36 | S. Rissen 6,7,14 f,g - | 8 |
| 37 | S. Rubislaw 11 r e,n,x | 7 |
| 38 | S. Augustenborg 6,7,14 i 1,2 | 7 |
| 39,40 | S. Agoueve or S. Cubana (share the same general formula: 13:$z_{29}$:-) | 7 |
| 41 | S. Hvittingfoss 16 b e,n,x | 6 |
| 42 | S. Braenderup 6,7,14 e,h e,n,$z_{15}$ | 6 |

Supplementary Table 1

Serovars serotyped in 1041 isolates (Continued):

| | *Salmonella* **serovar and its antigenic formula** | **Number of isolates** |
|---|---|---|
| 43,44 | S. Hindmarsh or S. Bovismorbificans (share the general formula: 8:r:1,5) | 5 |
| 45 | S. Caracas [1],6,14,[25] g.m.s - | 5 |
| 46 | S. Mgulani 38 i 1,2 | 5 |
| 47 | S. Liverpool 1,3.19 d e,n.$z_{15}$ | 5 |
| 48,49 | S. Pakistan or S. Litchfield (share the same general formula: 8:l,v:1,2) | 5 |
| 50 | S. Farmingdale IV 43:$z_4$,$z_{23}$:- | 5 |
| 51 | S. Tennessee 6,7,$\underline{14}$ $z_{29}$ [1,2,7] | 4 |
| 52 | S. Elomrane $\underline{1}$,9,12 $z_{38}$ - | 4 |
| 53 | S. Potsdam 6,7,$\underline{14}$ 1,y e,n,$z_{15}$ | 4 |
| 54 | S. Warragul [1],6,14,[25] g,m - | 4 |
| 55,56 | S. Virginia or S. Muenchen (share the same general formula: 8:d:1,2) | 4 |
| 57 | S. Eastbourne $\underline{1}$,9,12 e,h 1,5 | 4 |
| 58 | S. Idikan $\underline{1}$,13,23 i 1,5 | 4 |
| 59 | S. Reading 1,4,[5],12 e,h 1,5 [R1…] | 4 |
| 60 | S. Wandsworth 39 b 1,2 | 3 |
| 61,62 | S. Emek or S. Chincol (share the same general formula: 8,$\underline{20}$ g,m,s - ) | 3 |
| 63 | S. Perth 38 y e,n,x | 3 |
| 64 | S. Kumasi 30 $z_{10}$ e,n,$z_{15}$ | 3 |
| 65,66 | S. Kottbus or S. Ferruch (share the same general formula: 8:e,h:1,5) | 3 |
| 67,68 | S. Glostrup or S. Chomedy (share the same general formula: 8:$z_{10}$:e,n,$z_{15}$) | 3 |
| 69 | S. Ealing 35 g,m,s - | 3 |
| 70 | S. Zigong 16 1,w 1,5 | 3 |
| 71 | S. Abony $\underline{1}$,4,[5],12,$\underline{27}$ b e,n,x | 3 |
| 72 | S. Kirkee 17 b 1,2 | 3 |
| 73 | S. London 3,{10}{15} 1,v 1,6 | 3 |
| 74 | S. Oslo 6,7,$\underline{14}$ a e,n,x | 3 |
| 75 | S. Teko [1],6,14,[25] d e,n,$z_{15}$ | 3 |
| 76 | S. Give 3,{10}{$\underline{15}$}{$\underline{15,34}$}} 1,v [d] | 3 |
| 77,78 | S. Haardt or S. Blockley (share the same general formula: 8:k:1,5) | 2 |
| 79 | S. Cairina 3,10 $z_{35}$ $z_6$ | 2 |
| 80,81 | S. Albany or S. Duesseldrof (share the same general formula: 8:$z_4$,$z_{24}$:- ) | 2 |
| 82 | S. Vitkin 28 1,v e,n,x | 2 |
| 83 | S. Richmond 6,7 y 1,2 | 2 |
| 84 | S. Carmel 17 1,v e,n,x | 2 |
| 85 | S. Kokomlemle 39 1,v e,n,x | 2 |
| 86 | S. Livingstone 6,7,$\underline{14}$ d 1,w | 2 |

Supplementary Table1

Serovars serotyped in 1041 isolates (Continued):

| | ***Salmonella* serovar and its antigenic formula** | **Number of isolates** |
|---|---|---|
| 87 | S. Bangkok 38 $z_4$,$z_{24}$ - | 2 |
| 88 | S. Sandiego 1,4,[5],12 e,h e,n,$z_{15}$ | 2 |
| 89 | S. Plymouth 9,46 d $z_6$ | 2 |
| 90 | S. Abaetetuba 11 k 1,5 | 2 |
| 91 | S. Amsterdam 3,{10}{15}{15,34} g,m,s - | 2 |
| 92 | S. Szentes 16 k 1,2 | 2 |
| 93 | S. Yoruba 16 c 1,w | 2 |
| 94 | S. Ball 1,4,[5],12,27 y e,n,x | 2 |
| 95 | S. Indiana 1,4,12 z 1,7 | 2 |
| 96 | S. Lingwala 16 z 1,7 | 2 |
| 97 | S. Pomona 28 y 1,7 [$z_{80}$],[$z_{90}$] | 2 |
| 98 | S. Ruiru 21 y e,n,x | 2 |
| 99 | S. Johannesburg 1,40 b e,n,x | 2 |
| 100 | S. Telaviv 28 y e,n,$z_{15}$ | 2 |
| 101 | S. Colinade 6,7 r 1,7 | 2 |
| 102 | S. Matopeni 30 y 1,2 | 2 |
| 103 | S. Putten 13,23 d 1,w | 2 |
| 104 | S. Vancouver 16 c 1,5 | 2 |
| 105 | S. Chester 1,4,[5],12 e,h e,n,x | 2 |
| 106 | S. Butantan 3,{10}{15}{15,34} b 1,5 | 1 |
| 107 | S. Galiema 6,7,14 k 1,2 | 1 |
| 108 | S. Gbadago 3,{10}{15} c 1,5 | 1 |
| 109 | S. Salford 16 1,v e,n,x | 1 |
| 110 | S. Tamilnadu 6,7 $z_{41}$ $z_{35}$ | 1 |
| 111,112 | S. Corvallis or S. Chailey (share the same general formula: 8:z4,z23:-) | 1 |
| 113 | S. Halle 28 c 1.7 | 1 |
| 114 | S. Cleveland 6,8 $z_{10}$ 1,7 | 1 |
| 115 | S. Ilala 28 k 1,5 | 1 |
| 116 | S. Lindenburg 6,8 i 1,2 | 1 |
| 117 | S. Adelaide 35 f,g - [$z_{27}$] | 1 |
| 118 | S. Nima 28 y 1,5 | 1 |
| 119 | S. Ank 28 k e,n,$z_{15}$ | 1 |
| 120,121 | S. Soahanina or S. Sundsvall (share the same general formula: 6,14:z:e,n,x ) | 1 |
| 122 | S. Luke 1,47 g,m - | 1 |
| 123 | S. Simi 3,10 r e,n,$z_{15}$ | 1 |

Supplementary Table 1

Serovars serotyped in 1041 isolates (Continued):

| | ***Salmonella* serovar and its antigenic formula** | **Number of isolates** |
|---|---|---|
| 124,125 | S. Takoradi or S. Bargny (share the same general formula: 8:i:1,5 ) | 1 |
| 126,127 | S. Diguel or S. Telelkebir (share the same general formula: 13:d:e,n,$z_{15}$ ) | 1 |
| 128 | S. Baguida 21 $z_4$,$z_{23}$ - | 1 |
| 129 | S. Nchanga 3,{10}{15} 1,v 1,2 | 1 |
| 130,131 | S. Carrau or S. Madelia (share the same general formula: 6,14:y:1,7 | 1 |
| 132,133 | S. Miami or S. Sendai (share the same general formula: 9:a:1,5) | 1 |
| 134 | S. Llandoff 1,3,19 $z_{29}$ [$z_6$] [$z_{37}$] | 1 |
| 135 | S. Mikawasima 6,7,14 y e,n,$z_{15}$ [$z_{47}$],[$z_{50}$] | 1 |
| 136 | S. Odozi 30 k e,n,[x],$z_{15}$ | 1 |
| 137,138 | S. Kintambo or S. Washington(share the same general formula: 13:m,t) | 1 |
| 139 | S. Nottingham 16 d e,n,$z_{15}$ | 1 |
| 140 | S. Singapore 6,7 k e,n,x | 1 |
| 141 | S. Soerenga 30 i 1,w | 1 |
| 142,143 | S. Hadar or S. Istanbul (share the same general formula: 8:$z_{10}$:e,n,x) | 1 |
| 144 | S. Elisabethville 3,{10}{15} r 1,7 | 1 |
| 145 | S. Hato 1,4,[5],12 g,m,s [1,2] | 1 |
| 146 | S. Barmbek 16 d $z_6$ | 1 |
| 147 | S. Kedougou 1,13,23 i 1,w | 1 |
| 148 | S. Tarshyne 9,12 d 1,6 | 1 |
| 149 | S. Durban 1,9,12 a e,n,$z_{15}$ | 1 |
| 150 | S. Altona 8,20 r,[i] $z_6$ | 1 |
| 151 | S. Welikade 16 1,v 1,7 | 1 |
| 152 | S. Heidelberg 1,4,[5],12 r 1,2 | 1 |
| 153 | S. Alachua 35 $z_4$,$z_{23}$ - [$z_{37}$],[$z_{45}$] | 1 |
| 154 | S. Weslaco 42 $z_{36}$ - | 1 |
| 155 | S. Mountpleasant 47 z 1,5 | 1 |
| 156 | S. Minnesota 21 b e,n,x [$z_{33}$],[$z_{49}$] | 1 |
| 157 | S. Urbana 30 b e,n,x | 1 |
| 158 | S. Stormont 3,10 d 1,2 | 1 |
| 159,160 | S. Lindern or S. Charity (share the same general formula: 6,14:d:e,n,x ) | 1 |
