# Supplementary material for: Salmonella Serotyping Using Whole Genome Sequencing
Source: Front Microbiol. 2018 Dec 13;9:2993. doi: 10.3389/fmicb.2018.02993 (PMC6300517; doi:10.3389/fmicb.2018.02993)
Supplement: Supplementary file 2 [file Table_2.PDF]

Supplementary Table 2: showing traditional serology results of 62 isolates which could not be assigned by SeqSero

Accession ID #	traditional serotyping results
29	*Salmonella* Tarshyne
44	*Salmonella* Group C2, No 1st phase flagellar antigen
47	*Salmonella* Degania
60	*Salmonella* Group E1, incomplete flagellar antigen
72	*Salmonella* Florian
73	Isolate non-viable
138	*Salmonella* Subspecies 2 Group 41
139	*Salmonella* Group 30
224	*Salmonella* Bareily Group C1
225	*Salmonella* Nchanga Group E1
284<br>341	*Salmonella* Virchow Group C1
286	*Salmonella* ssp. 16,7:y:1,2,5 Group C1
287<br>701	*Salmonella* Newport
285<br>288<br>559<br>1048	*Salmonella* Montevideo Group C1
332	*Salmonella* Rissen Group C1
345	*Salmonella* Banjul Group H
356	*Salmonella* Wentworth Group F
372	*Salmonella* Group T ssp. VI (Indica) antigenic formula 42- Unable to further serotype.
416	*Salmonella* Group E1. Incomplete flagellar antigen
441<br>558	*Salmonella* Arizonae (s. IIIa-:r:-)
480	*Salmonella* Mbandaka Group C1
488	*Salmonella* Aberdeen group F
490	*Salmonella* Houten Group U
553	*Salmonella* Senftenberg Group E4
576	*Salmonella* Baguida Group L
597	*Salmonella* Group Q
610	*Salmonella* Harmelen Group 51
619	*Salmonella* Hagenbeck Group Y

Supplementary Table 2 (Continued): showing traditional serology results of 62 isolates which could not be assigned by SeqSero

**Accession ID #**	**traditional serotyping results**
304<br>701<br>703<br>787	*Salmonella* Gaminara Group I 16 d 1,7
763<br>777	*Salmonella* Subspecies IV serotype: 21:z36:-
372<br>834	*Salmonella* Subspecies VI (Indica). 9,46 1.v 1,5
812<br>827	*Salmonella* Group F Subspecies: 11:z4,z23:-
841<br>898	*Salmonella* Group Z antigenic formula =50:y :-
875	*Salmonella* Waycross Group S 41 z4,Z23 [e,n,z15]
880<br>884	*Salmonella* Dortmund Group E1 3,10 z41 1,[2],5
889	*Salmonella* Group C1 antigenic formula 16,7:i:-<br>Unable To further dtect.
895	*Salmonella* Monophasic Group C1
963	*Salmonella* Gbadago Group E1 3,{10}{15} c 1,5
179<br>977	*Salmonella* Tennessee Group C1 6,7,14 z29 [1,2,7]
1047	*Salmonella* Richmond Group C1 6,7 y 1,2
1049	*Salmonella* Schleissheim Group B 4,12,27 b -
1077<br>1078	*Salmonella* Enterica Subspecies Salame Group S<br>S. ssp. II 41:z10:1.2
1031<br>1093	*Salmonella* Adelaide Group O 35 f,g -
968	*Salmonella* Degania 401, 402: z4, z24: z39.
881	*Salmonella* Group C1 Antigenic formula=6,7:r:-
